# A combined qualitative–quantitative fuzzy method for urban flood resilience assessment in Karaj City, Iran

**DOI:** 10.1038/s41598-023-27377-x

**Published:** 2023-01-05

**Authors:** Kousha Khatooni, Farhad Hooshyaripor, Bahram MalekMohammadi, Roohollah Noori

**Affiliations:** 1 Department of Civil Engineering, Architecture and Art, Science and Research Branch, Islamic Azad University, Tehran, Iran; 2 School of Environment, College of Engineering, University of Tehran, Tehran, Iran

**Keywords:** Environmental social sciences, Hydrology, Natural hazards

## Abstract

This study aims to analyze flood resilience (FR) in Karaj City, Iran, using a new fuzzy method which combines several qualitative and quantitative indices. The qualitative part was estimated by a questionnaire consisting of 42 questions distributed into five indices (social-cultural, economic, infrastructural-physical, organizational-institutional, and hydraulic). A fuzzy method was used for analyzing the results. To quantify the hydraulic index, a 25-year flood was simulated in the Storm Water Management Model and the flooding volume at every grid was estimated. The idea was that the flooding amount could be representative of structural FR of drainage network that cannot be evaluated through a questionnaire well. To calculate the FR of different districts, the obtained FR indices were fuzzified then aggregated. Considering that clustering can assist managers and decision makers for more effective flood risk management, a fuzzy equivalence matrix concept was used for clustering FR in the city. Friedman test showed the significance of differences between FR of every two districts. Based on the results, northwestern and southeastern districts had the highest and the lowest resilience, respectively. Although the impact of infrastructure-physical index on the FR was similar in most of the districts, the contribution of social-cultural, organizational-institutional, and hydraulic indices was significantly different. Also, districts with low scores in the infrastructure-physical, organizational-institutional, and hydraulic indices need more attention for flood risk management.

## Introduction

In recent decades, many countries around the world have experienced natural disasters such as floods, tsunamis, storms/hurricanes, and earthquakes and received much damage in urban systems^1^. Despite the complexity of natural disasters prediction, many countries have targeted disaster mitigation strategies^2,3^. Although there are some methods to mitigate the adverse effects of natural disasters, humanity cannot prevent them all. Therefore, the capacity of resistance (associated with processes that enable the tolerance of, or adaptation to, a disturbance^4^) and resilience (associated with recolonization, reproduction, or rapid regrowth^4^) of residents against these events should be improved^5^. Among all natural disasters, the most economic, social, and environmental consequences have been reported because of flooding events^1^. Meanwhile, urban floods can create many problems in residential areas, although they could be managed through the concept of resilience^6^. This concept was first introduced by Holling^7^ in the field of ecology and environment. He defined the resilience of an ecosystem as the measure of its ability to absorb changes and still exist. Then, Bruneau et al.^8^ applied this concept to natural disasters such as earthquakes and floods. Flood resilience (FR) is defined as preserving cities from being flooded and the process of recover the flooded areas^5,9,10^. FR is minimizing the negative effects of floods before, during, and after a flood in terms of socio-ecological, economical, organizational, infrastructural, and hydraulic issues^11^. It means the ability of an urban system to recover from a shock and maintain its identity^11^. According to Zhong et al.^12^, because of low resilience, many residential areas are not able to quickly return to normal condition after a flood event; therefore, economic damage and human loss may intensify.

Although it is not possible to prevent all residential areas from being flooded, it is possible to increase their resilience. Resilience influences by different factors such as attitude, perception, and behavior of residents about disasters^9^. Elaborating FR can affect economic, personal, and social factors and improve the infrastructural, services, and managerial dimensions^5^. Li et al.^13^ showed that FR can improve the performance of water drainage systems by predicting the weak points; however, it can work as an improvement criterion for decreasing damage in urban areas. Wang et al.^14^ used the FR concept joined with land use and social media data and spatial–temporal patterns of urban flooding to measure both physical infrastructure and human elements resilience. Exploring the resilience-based practices in Melbourne, Australia, FR as an interdisciplinary approach was used by Rogers et al.^10^. According to the results, social science, urban design, and environmental engineering techniques can be used to gain integrated insights into FR and urban livability. Wang et al.^15^ provided a benchmark for urban FR assessment which uses mean flood duration and considers flooded nodes to mitigate flooding. Li et al.^16^ noticed that urban FR combined with the factor analysis method can be used to evaluate urban water system problems. This method can be applied to analyze the resilience of buildings and infrastructure systems in short-term and long-term periods. Urban infrastructures like communication networks and medical service centers can be considered the main factors in improving the resilience of cities against floods^17^. Meteorological forecasting plays an essential role in the FR^18^. Karrasch et al.^19^ presented flood resilience rose as a management tool to promote harmonized action toward FR in European regions and beyond. Meanwhile, some researchers have provided the method of FR assessment by descriptive data, mathematical tools, or a combination of tem^20^. A literature review shows that only 10.3% of the previous researches combined the descriptive data from a questionnaire with mathematical methods^20^. Cai et al.^20^ used a fuzzy analytical hierarchy process (FAHP) with a questionnaire and showed that the FAHP is fair to make a quick and regional flood risk assessment. Ezeokoli et al.^21^ used a questionnaire, Pearson product moment calculation, and Z-test to evaluate the FR in Ogbaru, Nigeria, and manifested that by restoring or reconstructing the drainage system, their resilience would be increased. In order to explore the strengths and weaknesses of different types of toolkits in FR assessment, Jones^22^ proposed a subjectivity–objectivity continuum that classifies measurement approaches according to two core tenets (a) how resilience is defined and (b) how resilience is evaluated. Wu et al.^23^ evaluated the impact of the design and configuration of urban flood control programs in Zhangjiagang City, China, on the FR during heavy rainfall events. By defining resilience benefit they determined the unit annual average cost of a program combined with its resilience benefit.

In most of the above-mentioned studies, descriptive data (qualitative method) has been used for FR; although, some of the FR indicators can be estimated in more detail by a precise mathematical model (quantitative method). The hydraulic capacity and capability of drainage network to convey the flood flow, the volume of flooding, and time of flooding are among those indicators that are proposed in this paper to be evaluated in a quantitative approach instead of a common qualitative approach. On the other hand, several studies^13,23^ have shown the capability of the fuzzy method to classify critical areas based on a weighting strategy. Hence, this study benefits from fuzzy equivalence matrix method to cluster the different districts of Karaj City based on their FR values. This classical method has not been used for FR clustering, yet. This fuzzy clustering approach in addition to its simplicity helps to identify similar districts in terms of resilience for prioritization of the practical actions since FR improvement can be considered as a time, energy, and money consuming action which needs priorities. Therefore, a combination of SWMM (Storm Water Management Model)^24^ to the common qualitative FR assessment and fuzzy clustering of FR can be mentioned as innovations of this study.

The paper includes a methodology section which contains information about the study area and the method of urban FR assessment in Karaj City. Following this section, the results of the study and discussion parts are presented. This paper ends with a conclusion part and suggestions for further research.

## Methodology

### FR assessment framework

**Figure 1:**
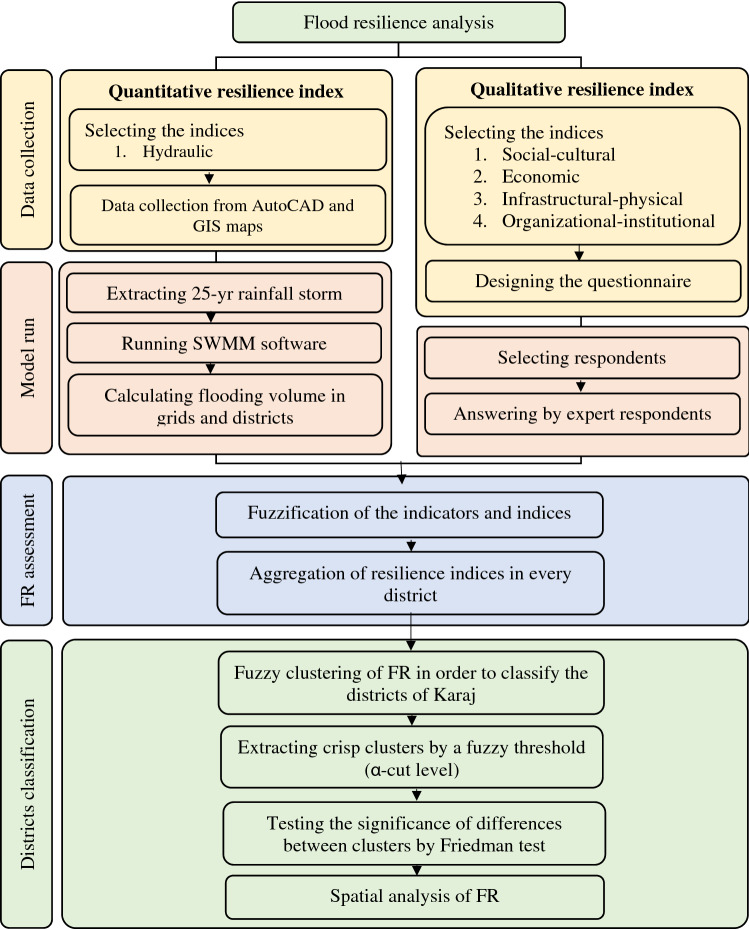
Summary of the methodology used in this study.

For evaluating FR in Karaj City, a combined new method was employed. On one hand, the hydraulic situation of Karaj’s drainage network was evaluated using SWMM model to identify the grids that have limited capacity for flood drainage. On the other hand, a questionnaire was designed to evaluate different effective factors on FR. Finally, a fuzzy approach was used to assess FR of different districts of Karaj and to cluster the districts according to their resilience. A summary of the research methodology can be found in Fig. 1.

According to Fig. 1, in order to study the resilience against urban flood in Karaj, 5 indices were defined: (1) social-cultural, (2) economic, (3) infrastructural-physical, (4) organizational-institutional, and (5) hydraulic (Table 1). The indices, extracted according to the literature^1,11,18,25–28^ and developed by the authors. Every index was quantified according to several indicators (Table 2).

**Table 1:** Various resilience indices with their quick definition.

Index	Operational definitions
Social-cultural	The amount of social and cultural capital according to variables of trust (institutional—public), cohesion and continuity, and existence of social networks and non-governmental organizations (relief participation)
Economic	This index is assessed on the basis of economic indicators such as living conditions, business and income, and satisfaction with salaries and life expenditures
Infrastructural-physical	This index is based on the sense of belonging to a desired place and neighborhood, level of satisfaction with neighborhood services and accessibility to them, and assessing the insurance of households. It includes indicators such as education, health, public transportation, cultural and business complexes, hospitals, strength of buildings, neighborhood security, and municipal services
Organizational-institutional	It shows the level of people's satisfaction with the relevant institutions in matters of information, relief and performance in the districts
Hydraulic	This index includes specialized hydraulic characteristics that assess the degree of clogging, volume of flooding, and performance of the water collection system during floods

**Table 2:** Details of the indices and indicators to assess FR in Karaj.

Index	Indicator
Social-cultural	1. What is the level of education in your family? 2. How much do you trust your neighbors? 3. Do you have easy access to health services? 4. Do you know any social groups in your district? 5. How do you assess the level of participation in social groups in your neighborhood? 6. Do you have insurance coverage (medical insurance, unemployment insurance)? 7. How do you assess your relationship with your neighbors? 8. Do you have easy access to social networks? 9. How do you assess the level of awareness about natural hazards in your family?
Economic	1. Do you have flood insurance? 2. Are you satisfied with your monthly income compared to the expenses? 3. Do you have any investment (housing; bank deposit; stock exchange; …)? 4. Do you have a second job or alternative income? 5. How vulnerable are your properties against flood? 6. How much money do you save every month?
Infrastructural-physical	1. How strong is your house? 2. Do you have easy access to welfare services such as a park, amusement, educational, and cultural? 3. What is the quality of your access to telephone, gas, water, and electricity infrastructure? 4. What is the population density in your region? 5. How many tall buildings are there in your region? 6. How much is the density of emergency fire stations in your region? 7. How is your access to hospitals and clinics in your vicinity? 8. How is your access to education centers in your neighborhood? 9. How is the situation of the road network in your neighborhood in terms of highways and streets? 10. How much are you satisfied with urban services?
Organizational-institutional	1. How do you assess the amount of information provided by the relevant organizations in the field of natural disasters and crisis management in your area? 2. How do you assess the performance of relevant organizations in the field of crisis management? 3. Are you satisfied with the administrative officials solving urban problems? 4. How active are volunteer groups in your region? 5. How is the level of public participation in your region? 6. What is your idea about the people's unity and solidarity in your region? 7. How many organizations relevant to crisis management are there in your neighborhood? 8. How is the availability of loans and bank grants for reconstruction after a crisis?
Hydraulic	1. How well is flood drained through the network in your area? 2. How old is the drainage network in your area? 3. Is usually clean the drainage network in your area? 4. Does the drainage network work well during rainfall events? 5. How many times have you observed obstruction and network collapse in your area? 6. Are there units in your area that dispose of their wastes/trash into the drainage network? 7. How satisfied are you with the performance of the drainage network during floods? 8. How many unpaved areas (green spaces and parks) are there in your area? 9. How steep is your area?

#### Quantitative resilience index

In this step, a mathematical model is employed for quantitative evaluation of the hydraulic index. Hydraulic characteristics of the network reflect the performance of drainage network during urban floods. In addition, lack of cleanliness of conduits and existence of trash in the sewer network during urban floods are among the problems that can enhance the flood severity^27,29^. Moreover, failed conduits can endanger the properties around the conduits by increasing the inundation depth. For instance, clogging in a main conduit could intensify the flood and incrementing the inundation areas. Indeed, an important part of urban FR refers to the ability of the drainage system which represents the structural resilience of the system against floods. Flooding in a network can be due to poor design and insufficient capacity of conduits. To consider this part of resilience, the amount of flood volume in different parts of the network is calculated and used as an indicator for the hydraulic resilience. Doing so, the SWMM model is used to determine the critical nodes and flooding amounts. Inputs of the model include characteristics of sub-catchments, conduits, meteorological information such as precipitation data, conceptual model information, and simulation method.

SWMM uses several loss models for instance, Horton, Green-Ampt, and soil conservation service (SCS) model which is based on curve number (CN). In SCS method, the effective rainfall (*P*_*e*_) is calculated as follows:

1 $$P_{e} = \frac{{\left( {P - I_{a} } \right)^{2} }}{{P - I_{a} + S}},$$

where *P* is rainfall (in); *S* is potential maximum retention equal to 1000/CN-10 (in), and *I*_*a*_ is initial losses. Based on SCS recommendation, the initial losses can be estimated as *I*_*a*_ = 0.2*S*^30^. SWMM solves the St-Venant equations (Eq. 2) for flood routing in the conduits.

$$\frac{\partial A}{{\partial t}} + \frac{\partial Q}{{\partial x}} = 0,$$

2 $$\frac{1}{A}\frac{\partial Q}{{\partial t}} + \frac{1}{A}\frac{\partial }{\partial x}\left( {\frac{{Q^{2} }}{A}} \right) + g\frac{\partial y}{{\partial x}} - g(S_{o} - S_{f} ) = 0,$$

where *x* is distance, *t* is time, *A* is cross section of flow, *y* is blue load, *S*_*0*_ is slope of conduit, *S*_*f*_ is frictional slope in the duct, and *g* is gravitational acceleration.

In the current investigation, SCS method was utilized to model the deep water percolation in the soil. As the rainfall data, a uniform 25-year rainfall was extracted from intensity–duration–frequency (IDF) curves that (are available from Taheri et al.^31^) and was put in the model. In addition, the characteristics of sub-basins, including slope, area, percentage of impermeable areas, and land uses were estimated using local usual AutoCAD maps (available from Karaj Municipality) and GIS maps (available from National Geographical Organization of Iran). Information about the canals and their dimensions was also obtained from Karaj Municipality.

#### Qualitative resilience index

In this paper, a questionnaire with 42 questions (indicators) was used to investigate five qualitative indices (Table 2). To do so, the questionnaire was completed by interviewing with several experts. For every question, the respondents have five options: (1) very low, (2) low, (3) medium, (4) good, and (5) very good. However, some of the answer options were changed from very low to very high according to the questions. For the evaluation of the social-cultural index, nine questions about social services, neighbor and family relations, and public awareness levels are gathered. For economic index evaluation, six questions were defined. The questions cover a wide range of indicators consisting of income, investment, insurance, property, and power saving behaviors of households. For the infrastructural-physical index, 10 questions that evaluate the level of structural strength, availability of emergency services, and accessibility to the public areas were prepared. The prepared questionnaire was given to be completed by 200 experts from water management companies, university professors and residents of each region to be used for assessment of FR in Karaj.

The authors tried to collect the information related to every district in a categorized manner. Therefore, resident samples of a district were selected randomly from employees in services, contractors, and consultant water related companies. Other samples were added to the list of respondents from the municipality and Water Supply Company of every district. The above samples were specific respondents to every district. On the other hand, some respondents were asked to fill out questionnaires for different districts that they were familiar with; specifically, professors of universities and employees from Alborz Water Company. It is worth noting that demographic questions such as background information of respondents such as age, level of education, and gender were asked by keeping their information confidential.

#### Questionnaire fuzzy analysis

To evaluate the questionnaires, a fuzzy method was used. In this method, respondents’ questionnaires for every district of Karaj were analyzed separately and the qualitative responses were standardized between 0 and 1 to be used for defining fuzzy sets according to Table 3.

**Table 3:** Fuzzy sets to assess the questionnaires.

Very poor	Poor	Fair	Good	Very good
(0, 0. 25)	(0,0.25, 0.5)	(0.25, 0.5, 0.75)	(0.5, 0.75, 1)	(0.75, 1)

Based on the frequency of choosing each of the options by different respondents for every indicator a fuzzy set is constructed. The generated membership functions are usually triangular, but if the results are scattered, a trapezoidal membership function is used. Then for every index, by aggregation of all the fuzzy sets of the different indicators, a resultant fuzzy set is obtained for that index. The same procedure is followed to evaluate the questions involved in the other indices. Then, there will be five fuzzy sets corresponding to the five indices specified in the questionnaires. Subsequently, with the center of area (*CoA*) defuzzification method, the crisp score of the FR indices is calculated (Eq. 3)^32^.

3 $$CoA=\frac{\underset{}{\overset{}{\int }}xf\left(x\right) dx}{\underset{}{\overset{}{\int }}f\left(x\right) dx},$$

where *f(x)* is membership function.

Finally, the average of all indices can be considered as the final FR for each district. This means that different indices with the same weight contribute to the final FR value.

### FR clustering

A fuzzy clustering approach using equivalence relations was employed for the classification of Karaj districts in accordance with FR. This method is based on a two-by-two similarity of data belonging to a set^32^. In this method, it is necessary to form a data similarity matrix based on the similarity criteria. The fuzzy relation *R* defined on the reference set *X* is called an equivalence relation if it has three properties: reflection, symmetry and alignment^33^. If the fuzzy relation *R* has only two reflective and symmetric properties, it is called a tolerance relation or fuzzy harmonizer. A tolerance or fuzzy harmonizer relation can be converted to an equivalence relation with a maximum of (*n *− 1) combination with itself^33^, where *n* is the number of data studied to form a similarity matrix (here the number of Karaj districts). Then the data form an array such as *X*. Each element of the *X* array itself forms a vector of length *m*. The elements of the similarity matrix are calculated by comparing the two data *x*_*i*_ and *x*_*j*_, and the intensity of similarity between them. The resulting matrix will be an *n* × *n* matrix. There are different methods for creating equivalent matrices; e.g., Max–Min and Cosine Amplitude methods. In the Max–Min method, the equivalence matrix elements are determined as follows^32^:

4 $${r}_{ij}=\frac{\sum_{k=1}^{m}\mathrm{min}\left({x}_{ik },{x}_{jk}\right)}{\sum_{k=1}^{m}\mathrm{max}\left({x}_{ik }, {x}_{jk}\right)},$$

where, *i*,*j* = 1,2,…,n. *r*_*ij*_ is a fuzzy value that ranges between 0 and 1. The closer *r*_*ij*_ to one, the greater the similarity between *x*_*i*_ and *x*_*j*_.

Having the equivalence matrix, the concept of *α*-cut is used to cluster the iso-resilient districts of Karaj. Using this method, different districts can be placed in the same clusters for a given *α*-cuts. This means that districts in a given cluster are similar in terms of FR and districts in different clusters are significantly different in terms of FR. An *α*-cut of a fuzzy set is a crisp set, which holds the elements of a fuzzy set (on the same universe of discourse) whose membership degree is greater than or equal to *α*^32^. Therefore, to have a defuzzified crisp equivalence relations, the elements of the fuzzy equivalence matrix greater than *α* are converted to one, and the others are changed to zero. By forming the crisp equivalence matrix, those columns that have the same mutual elements are placed in a cluster^32^.

### Friedman test

Friedman test was used to show the significance of the differences between 12 districts of Karaj City and their ranking in the case of resilience. This test is a non-parametric analysis in which the variance of repeated measures (within the groups) is calculated to compare the mean rankings among *k* variables (group)^34^. In other words, the null hypothesis (H_0_) is based on the same mean rank among the groups. Rejection of the null hypothesis means that at least two groups are significantly different. Accordingly, the following assumptions are considered in this test:

1. The same assumptions of variance or abnormality of data distribution are not observed.
2. The scale of the dependent variable should be at least rank.
3. There should be at least two groups.

In order to run the Friedman test, the researchers assumed the data matrix as {*x*_*ij*_}_*n*×*k*_* (n* as rows or blocks and *k* is the columns or treatment). It is worth mentioning that, the values of the observations in this matrix are the "Rank" value of each block or row. The average ranks for the *j* column (${\overline{\mathrm{r}} }_{.j}$) are calculated as follows^34^.

5 $${\overline{\mathrm{r}} }_{.j}=\frac{1}{n} \sum_{i=1}^{n}{r}_{ij}.$$

Having the average of the obtained scores, the Friedman test statistic (F), will be calculated as follows^34^.

6 $$F=\frac{12n}{k\left(k+1\right)} {\sum_{j=1}^{k}({\overline{\mathrm{r}} }_{.j}-\frac{k+1}{2})}^{2}.$$

With respect to Friedman test, if *k* or *n* has high values (for example, k ≥ 3 and n ≥ 15), *F* values approximately have a "Chi-squared distribution" with *k* − 1 degree of freedom. In this case, p-value will be measured as follows.

7 $$\mathrm{p}-\mathrm{value}=p\left({{x}^{2}}_{k-1}\ge \mathrm{F}\right),$$

If *k* and *n* have low values, the approximation will be weak and the probability value must be derived from the Friedman test tables. It is clear that, if the probability value is significant, the null hypothesis will be rejected, which indicates that the treatments are the same. On the other hand, if the calculated value of χ^2^ (*F*) is greater than or equal to the critical value of χ^2^ (p-value > 0.05), the null hypothesis is rejected. Therefore, in the directionless hypothesis, it can be concluded that with 95% confidence, the context of the dependent variable is different or the sum of the rankings is significantly different from each other.

### Case study

**Figure 2:**
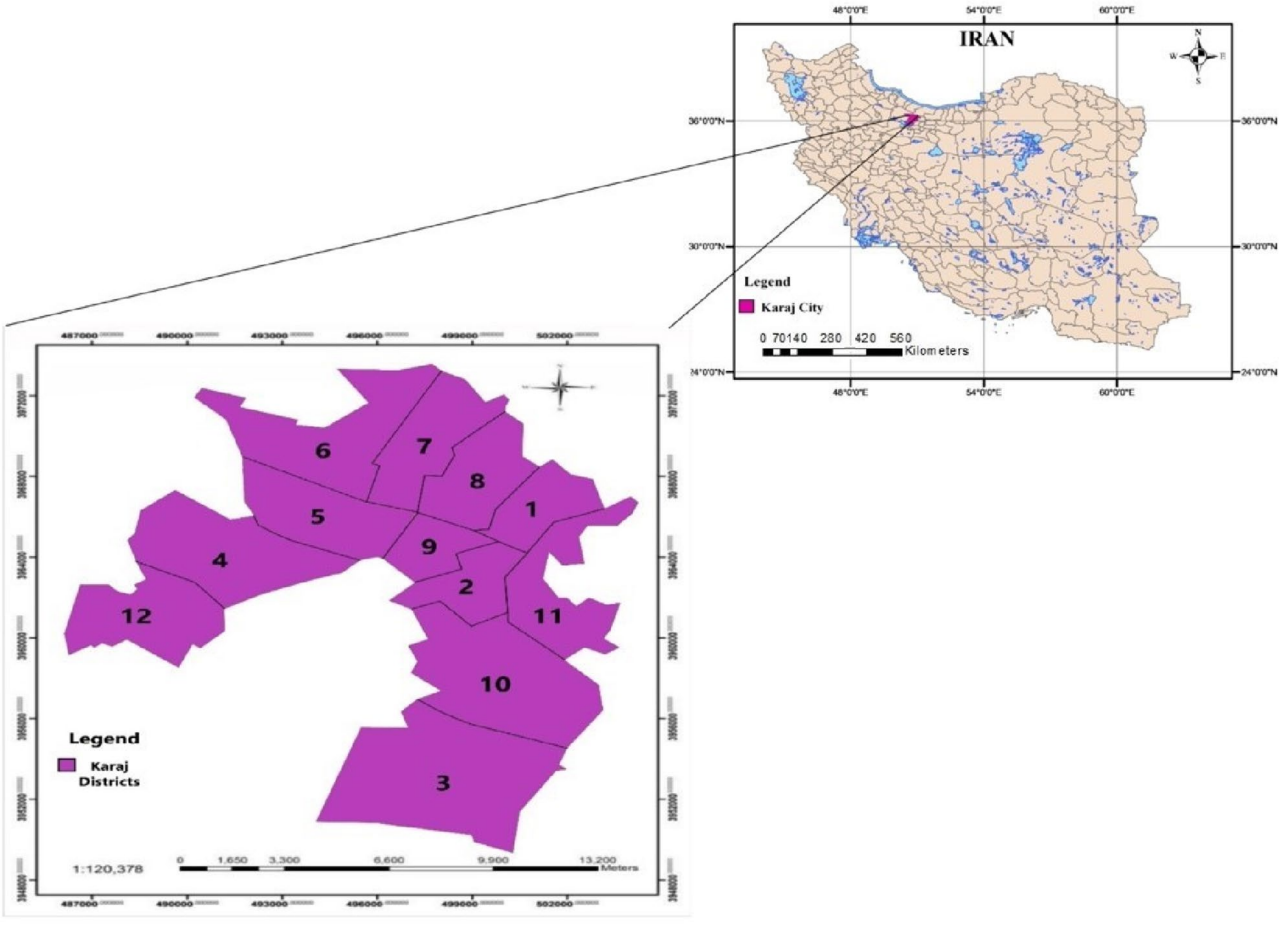
The geographical location of Karaj and its 12 districts in Iran (This figure was created in the Quantum GIS, version 3.12.2).

In this investigation, Karaj City, the center of Alborz Province (located in Iran), was selected as the area of study. Karaj is an important city because of its high and dense population, big area, and the historical urban floods in the past, as well. This city with an area of 175 km^2^ is located between latitudes 41° 35′ N and 53° 35′ N and between longitude 50° 50′ E and 51° 02′ E. Its average altitude is 1312 m above sea level and it is located in a mountainous area of Central Alborz Mountains (Fig. 2). It is bounded by the Alborz Mountains in the north and northeast. According to the digital elevation model, the average slope of the city is 5.5%. Karaj River with 245 km in length flows through the east of the city. The river’s average slope is about 0.8% and its average annual discharge is 499 mcm. In 1961, a large concrete dam (Amir-Kabir Dam) with a capacity of 205 mcm was built on the river for the water supply of Karaj and Tehran. The average annual precipitation of Karaj City is 247.3 mm. The temperature varies between − 20 and 42 °C. The annual average relative humidity is 52%. Rainfall in Karaj happens from almost January to April and November, and December. Because of overgrazing and deforestation attendant on the development of Tehran, the plant cover of this high mountain area is very sparse, increasing the danger of flood and soil erosion. The geographical location of the Karaj metropolis in the southern Alborz foothills is such that it is naturally exposed to floods with minimal rainfall. The Flood of July 2015 in Karaj killed 8 persons and inundated many buildings and roads^35^. On October 28th, 2019 flood interrupted the traffic in the city and interurban roads. The latest catastrophic urban flood occurred on July 22th, 2022 with a lot of damage to the properties^35^.

It is worth mentioning that the population of Karaj during the past two decades has grown rapidly by a 4.7% growth rate which ranks first in Iran^36^. Because of many industrial factories and working positions, it has attracted a large inland immigrant population. The rate of immigration in this city was about 7.7% from 1976 to 1986. Currently, the population of Karaj is over 1.6 million people with a density of about 9100 persons per square kilometer^36^.

## Results and discussion

### Hydraulic index

**Figure 3:**
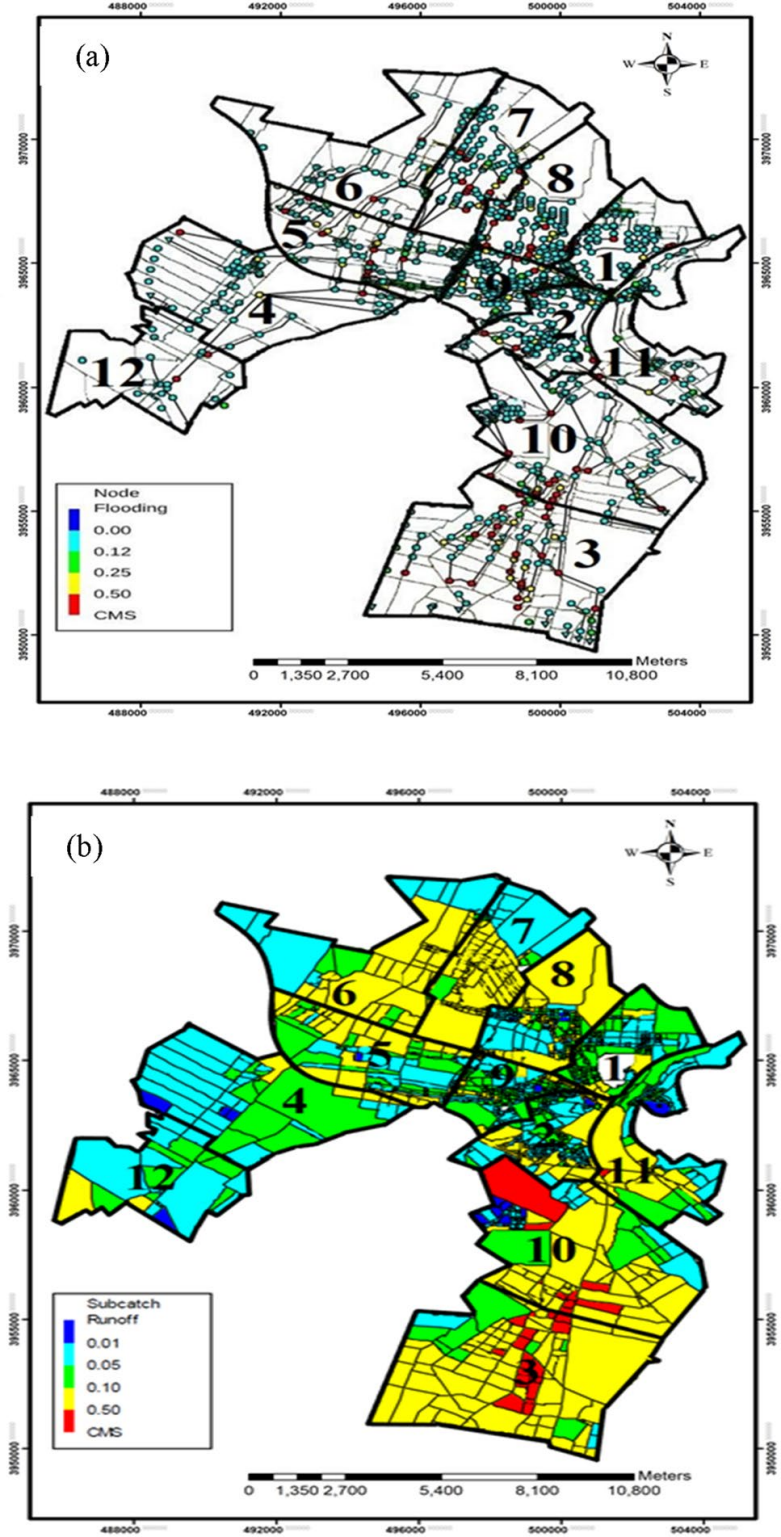
Map of (**a**) flooding in different nodes and (**b**) runoff in different sub-catchments (This figure was created in the Quantum GIS, version 3.12.2).

As mentioned earlier, the volume of flood over the network capacity is representative of the hydraulic index. In this study, flood volume is calculated in every node using SWMM software. The hydraulic model contains 919 nodes and 874 conduits for collecting urban runoff and 26 outlet nodes to discharge this volume of runoff. As the city is located at the foothills of Alborz Mountain, it receives upstream runoffs from 9 points in the north. Therefore, these inputs were calculated^31^ and applied in the upstream nodes. After entering the necessary information for a 25-year storm, the amount of flooding in different nodes and consequently its total in every district of Karaj were calculated. Figure 3a shows the flooding in every node and Fig. 3b shows the amount of runoff in different sub-catchments. In Fig. 3a, the blue marks indicate the flood-free and stable state and the red marks indicate a flooding state. According to the results of the SWMM model, 272 nodes are being flooded under the 25-year storm event. Most of the flood volume (294,326 m^3^) occurs in district #3, namely Fardis. Also, among different districts, the lowest flood volume happens in district #4, Mehr-Shahr, with a value of 21.01 m^3^.

Finding review and Fig. 3b show that district #3 has the worst condition of runoff compared to the other districts. The total runoff in this district cannot well be drained, because of the low capacity of drainage. It is worth noting that district #4 has been slightly flooded. In this regard, the best performance returns respectively to districts #4, #10, #7, #6, #8, and #11. A review of the findings from the changes in nodes manifests that rainfall plays a very crucial role in flooding nodes. The volume of flooding in every district is presented in Table 4. These values will be used as one of the essential indicators in the FR analysis in Karaj.

**Table 4:** The amount of flooding in different districts of Karaj.

District	Number of nodes	Number of flooded nodes	Flood volume (10^3^ m^3^)
#1	79	17	285.88
#2	107	25	81.33
#3	81	48	294.32
#4	52	12	21.03
#5	49	18	158.99
#6	39	10	125.07
#7	97	28	224.16
#8	101	32	235.72
#9	118	20	178.34
#10	87	31	148.74
#11	76	20	208.04
#12	33	11	27.30
Total	919	272	1988.94

### Questionnaire analysis

The questionnaire was distributed in different districts of Karaj and completed by a total of 200 experts. The responses were evaluated separately for every district. In Table 5, the values of resilience indices which are calculated using Eq. (3) are provided. From Table 5 one can find the district which is the most resilient and the most unstable one. It can be seen that district #4 (Mehr-Shahr) has the most FR and district #11 shows the weakest performance against floods.

**Table 5:** Resilience indices in different districts and final FR values.

District	Index					FR	Rank
	Social-cultural	Economic	Infrastructural-Physical	Organizational-institutional	Hydraulic		
1	0.61319	0.5252	0.6786	0.4512	0.5429	0.56221	2
2	0.5899	0.4663	0.5643	0.4501	0.5054	0.51520	9
3	0.5880	0.4664	0.5590	0.4688	0.4481	0.50606	10
4	0.6336	0.5010	0.6161	0.4970	0.6097	0.57148	1
5	0.5866	0.5228	0.5208	0.4874	0.4968	0.52288	5
6	0.5648	0.4829	0.5603	0.4896	0.4757	0.51466	7
7	0.6265	0.5579	0.7319	0.4644	0.4501	0.56616	4
8	0.6218	0.5310	0.6532	0.4760	0.4815	0.55270	3
9	0.5840	0.4925	0.5013	0.4856	0.5323	0.51914	6
10	0.5399	0.4690	0.4724	0.5280	0.5114	0.50414	8
11	0.5671	0.4660	0.4542	0.4531	0.4810	0.48428	12
12	0.5562	0.4643	0.4833	0.4687	0.5113	0.49676	11

Friedman test then was applied to test the differences among the districts’ FR. The test was run in the SPSS software and the result is shown in Table 6.

**Table 6:** Result of Freidman test statistics.

Friedman statistic (F)	20.508
Degree of freedom	11
p-value	0.039

According to Table 6, as the p-value is less than 0.05, FR in the 12 districts of Karaj is significantly different.

**Figure 4:**
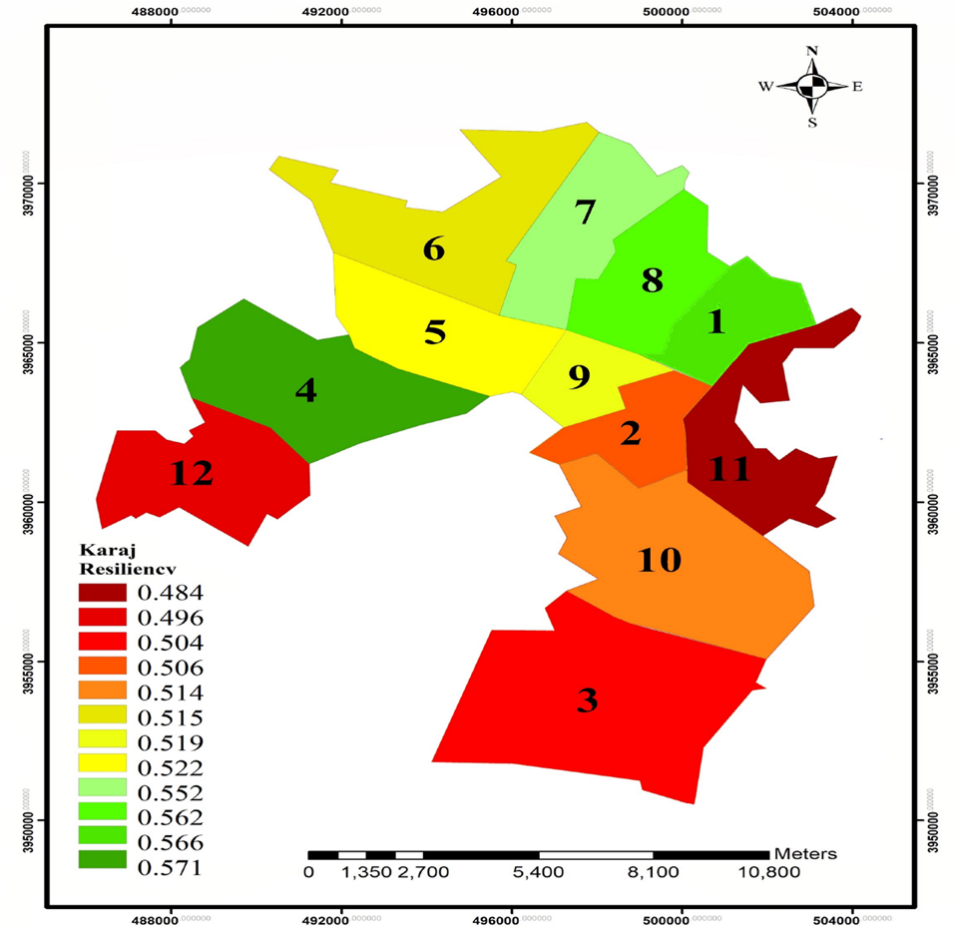
FR zoning in Karaj City (this figure was created in the Quantum GIS, version 3.12.2).

Analysis of the questionnaires shows some differences in the indices among the districts. For example, considering the hydraulic index, it is clear that the districts are significantly different, which means that districts that have more urban facilities and infrastructure, they have higher ranks. Figure 4 shows spatial variation of FR in Karaj. According to the figure, the northern districts, located at upstream, have a better FR situation and the southern districts have a worse situation.

### FR fuzzy clustering

The equivalence matrices method was used for the fuzzy clustering of different districts. To do this, using the max–min method, similarity matrices of 12 districts are created according to the score of different resilience indices. Table 7 presents the obtained similarity (equivalence) matrix for the Karaj districts by the maximum-minimum method. Equivalent districts are areas that are similar in terms of FR. Here, using the concept of *α*-Cut, the equivalent districts are identified. In the current study, *α*-Cut is considered to be 0.9. Table 8 shows the similarity matrix. In this table, the similar districts are shown in the same color.

**Table 7:** Equivalence matrix for Karaj districts based on Maximum-Minimum method.

Regions	1	2	3	4	5	6	7	8	9	10	11	12
1	1.0000											
2	0.9160	1.0000										
3	0.8876	0.9239	1.0000									
4	0.9197	0.9276	0.9256	1.0000								
5	0.9008	0.9362	0.9145	0.9248	1.0000							
6	0.8910	0.9227	0.9125	0.9307	0.9259	1.0000						
7	0.9220	0.8755	0.8567	0.8036	0.8731	0.8629	1.0000					
8	0.9385	0.9160	0.8026	0.9451	0.9103	0.8087	0.9138	1.0000				
9	0.8602	0.8018	0.8052	0.8014	0.9283	0.8986	0.8447	0.8743	1.0000			
10	0.8587	0.8023	0.8066	0.8939	0.9177	0.8995	0.8367	0.8727	0.9166	1.0000		
11	0.8463	0.8891	0.8963	0.8732	0.8084	0.8928	0.8160	0.8535	0.8099	0.8867	1.0000	
12	0.8720	0.9209	0.8091	0.9032	0.9282	0.8048	0.8455	0.8839	0.9247	0.9232	0.9110	1.0000

**Table 8:**
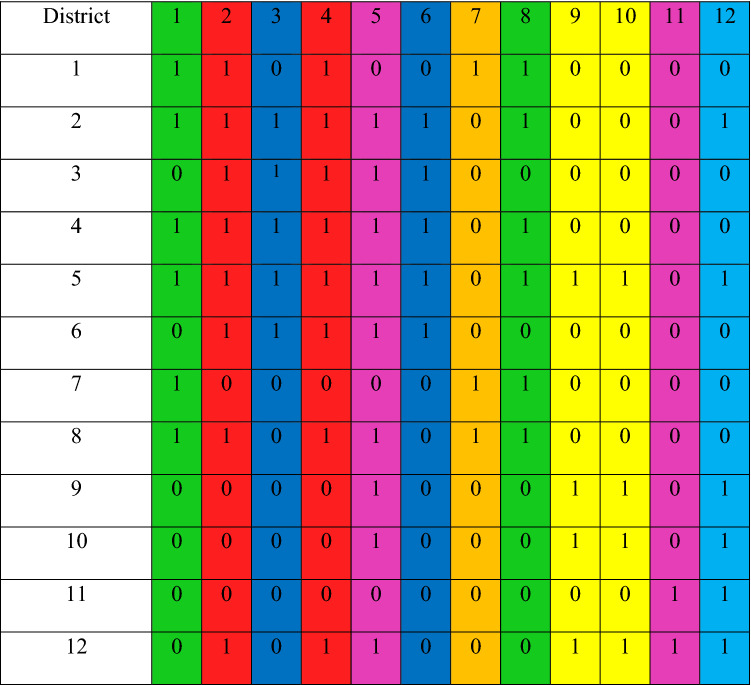
Equivalence matrix and clustering of Karaj City districts in terms of FR for *α*-Cut = 0.9.

**Figure 5:**
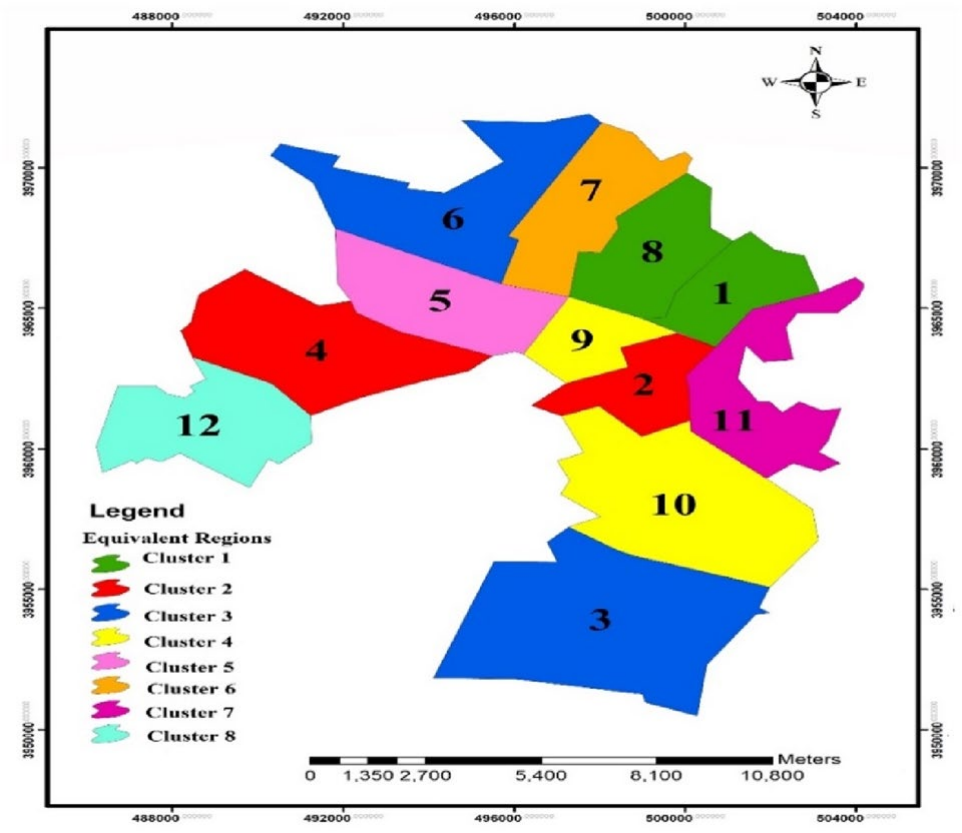
Crisp clustering map of FR in Karaj City for *α*-Cut = 0.9 (this figure was created in the Quantum GIS, version 3.12.2).

In the above equivalence matrix, the city of Karaj can be identified in 8 clusters. According to the results of Table 8, districts #1 and #8 are in the same cluster. Districts #2 and #4 are in the same cluster and are similar to each other. Also, districts #3, #6, #9, and #10 are in the same cluster. Other districts, which are not similar to the others, are considered unique clusters. Figure 5 shows the flood clustering map of Karaj City for *α*-Cut of 0.9.

According to Fig. 5, districts located in a cluster are similar in terms of indices defined for FR. For example, for *α*-Cut = 0.9, districts #3 and #6 have similar scores in economic and socio-cultural indices as well as physical and infrastructure indices. Those districts that are alone in a cluster have no similarity in the indicators with the other districts.

## Discussion

In order to discuss the findings of the study, it should be mentioned that most of the areas of Karaj City has the problem of flooding. Meanwhile, old districts and worn-out areas are more prone to flooding in the case of heavy precipitation. This result confirms the high potential of flood risk in the worn-out districts that was mentioned by Asadi et al.^37^, who provided strategies for urban infrastructure, facilities and equipment to be sustainable.Considering that global warming can intencify the flood consequences^9^ and cause unusual impacts on the natural events^38^, improving the FR in the urban areas becomes more important. Also, the results are in line with Moradpour et al.^39^ who stated informal settlements, worn-out urban fabrics, and central parts of cities have lower resilience to flood. The obtained results of our study showed that indices like economic and infrastructural-physical indices in most of the areas had a similar situation. Indeed, optimal layout of low impact development strategies^29^ can enhance the FR, especially in the old worn-out urban fabrics. In contrary, other indices like social-cultural, organizational-institutional and hydraulic were significantly different in different districts of Karaj which confirms the findings of Mousavi and Saadatmand^27^. Since the results showed that districts #1, #4, #7, and #8 had better performance, it can be said that these districts are more resilient than the others. However, still FR needs to be improved in these districts to enhance safety. Pouresmaeel et al.^40^ and Fekete et al.^41^ also mentioned the importance of FR in Karaj City. It should be noted that about 200 surveys were conducted in this research, that is, an average of 16 people were interviewed in each district. Although there are not many potential responders involved in urban flooding, more reliable results can be obtained by increasing the number of respondents. As mentioned in the last step of the questionnaire’s fuzzy analysis, all the indices were aggregated with the same weight, while their impact on the FR may be different. Therefore, it is suggested to evaluate the weight of the indices in a scientific manner to have more robust FR results in another study. One of the main limitations of this research was the lack of validation of the FR results. Because currently there is no systematic tool to record flood events and related damages on a district scale. For this reason, a complementary research can be very useful to validate the results. Despite examining just one design flood and all the aforementioned limitations, the authors propose this idea to be followed in further studies. In addition, it is suggested that the statistical population should be conducted with more samples so that the information obtained from this article can be examined and compared with them.

## Conclusions

Nowadays, FR is one of the concepts that has received much attention in flood management studies. To evaluate FR and identify the effective components of flood risk, the city of Karaj with 12 districts was considered as the study area. For this purpose, a questionnaire was prepared and distributed among 200 experts involved in flood engineering and residents of different districts. In the questionnaire, various social-cultural, economic, infrastructural-physical, organizational-institutions, and hydraulic indices were considered. In order to better investigate the hydraulic index, flooding volume in different districts was calculated using SWMM software. Then, composing the quantitative results of the SWMM model and qualitative outputs of the questionnaire, the final FR values of the districts were calculated. Based on the scientific recommendations^16,42^, this study used a fuzzy method for clustering FR in different districts of Karaj. The equivalence matrix method was employed for fuzzy clustering of FR, then using the *a*-cut concept the 12 districts of Karaj were arranged in 8 clusters. This clustering approach can help managers to devote priority to low quality districts for improving the resilience of the city. As a result, the resilience concept and fuzzy clustering could be effective in improving the FR of the districts. In general, the following results were obtained:

1. Investigation of flood in different districts showed that most areas suffer from flood and inundation, and in the meantime, older districts and worn-out areas that have an older drainage network are in a more critical situation.
2. In the study of the resilience questionnaire, it was found that economic and infrastructural-physical indices in most of the districts had a similar situation, while social-cultural, organizational-institutional, and hydraulic indices were significantly different.
3. The study of resilience promotion priority showed that districts with low infrastructural-physical, organizational-institutional and finally hydraulic indices, are among the most important urban areas that need priority for flood management.
4. The flood in the city of Karaj showed that the more resilient areas are #1, #4, #7, and 8 and these areas have a similar situation to each other and have been able to show better performance than other areas.
5. Examining the fuzzy clustering, it is clear that some of these areas have acquired similar status and behavior in resilience indices and their performance in FR is the same. For example, district #2 and #4 are located in one cluster and are similar and equivalent in all resilience indices.

## Data Availability

The datasets generated during and/or analyzed during the current study are available from the corresponding author on reasonable request.
